# Case of Inflammation in the Maxillary Sinus

**Published:** 1803-05-01

**Authors:** John King

**Affiliations:** Surgeon, of Walcot Terrace, Bath


Case *of* Inflammation *in the* Maxillary Sinus; *communicated by Mr.* John King, *Surgeon, of Walcot Terrace, Bath.*

Daniel Newton, the subject of this local affection, is twenty-six years of age, of a light complexion, sandy hair, dilated pupil, of an irritable habit, such as one might in other respects consider as a subject of latent scrophula; he had been for several years a private in the Essex light dragoons, and when I was assistant surgeon in that regiment some years ago, was under my care at Harlow Bush Common, where he received a kick on his leg from a horse, and though the wound was not large or the contusion great, still it proceeded very untowardly, (though the patient was under strict medical regime) which indicated the nature of his habit, and serves to shew the permanency of scrophulous diathesis, under whatever change of external circumstances, so that in this instance powerful anti-pyrexial tonic remedies, bandage, and horizontal posture were required to produce the healthy granulation. You will excuse this digression, as it serves to shew the constitutional diagnosis of our patient.

Since the period above alluded to, he had been ordered with the regiment to Ireland during the rebellion, and this young man was frequently on duty in very inclement weather, which, together with the debility induced by a want of salutary rest, exposed him to repeated defluxions in the head. The rheum from the head, in consequence of such attacks, was copious and frequent, and at length the mucous membrane of the nostrils became considerably inflamed. The nasal duct partook of the affection, so as to occasion some slight epiphoral discharge, which always receded on the amelioration of his health; afterwards the mucous membrane, which lines the cavity of the sinus, became inflamed, painful and distended, and lastly injured the alveolar process, decayed the tooth, and forced a discharge through the socket; but nothwithstanding that, the matter would often find its way into the nostril, and drop down by the side of the inferior spongious bone, so that from the subsequent account it should seem, from the long continued obstruction, that the matter had affected the surrounding bones of the sinus. The expedient of extracting the tooth subjacent was strongly pointed out and performed, but did not afford a free egress to the discharge, so that afterwards, on his first application to me, I thought proper proper to extract the tooth next in order, that the matter might have no obstruction to its descent; this step alleviated the disorder considerably. The pain, the distention, the external and integumental tumefaction and discoloration vanished. The egress to the discharge was uninterrupted, yet on a powerful inspiration it could be drawn into the nose and behind the velum pendulum palati. The discharge was very disagreeable, it dissolved the silver of the probe and turned it quite black, was very nauseating to the patient, and it was evident from the pallid, wasting and dyspeptic state he was reduced to, that its effects on the habit were morbidly increasing.

He assured me he had no local remains of any venereal complaint (unless this might be considered as such) nevertheless I judged proper to stimulate the system with bark and mercury, using by way of lotion the aq. zinci. vitriol. camphor. diluted, and drinking lime water, by which means he soon felt a sensible improvement in his health; the discharge diminished, became less offensive, but did not entirely cease.

With a very attenuated probe, which I gave the patient to insert at the aperture in the socket, he could make it pass directly into the nose, and by taking a fluid into his mouth and exhausting his mouth of air, the fluid could be made to pass in at the alveolar aperture and distil through the nose. This effort I desired him to repeat very often with lime-water, common water, or diluted vitriolic water with camphor, which, by the repeated strong perflations of the air through the aperture and sinus into the nostril, at length dislodged a large tuberous portion of bone, black and fetid; it was with difficulty the patient could discharge it by the mouth. I presume the fragment was of the maxillary bone, from its substance, (which, together with the lamella of the palate and part of the descending process of the sphenoid bones, form the parietes of the sinus.)

A long time had elapsed before any amendment took place, and the surgeon under whose care he was, offered no assistance but the bare extraction of the tooth, applied no constitutional remedies; and therefore, with what hopes could parts endued with extremely limited vitality, expect to derive advantage from their own feeble efforts, and probably labouring under specific morbid action; it is clear that the unassisted local impulsions of parts, remote from the energies of the habit, are inefficient to the speedy expulsion of parts become diseased and extraneous I have little doubt that, particularly during sleep, as the matter gravitated gravitated towards the throat and œsophagus, that the first morbid impressions were conveyed to the stomach, probably by an operation analogous to capillary attraction, or by simple absorption, or both conjointly; indeed, the sympathies of the stomach were aroused by the mortification of a large portion of bone pent up in the vicinity of the throat.

The effluvia itself was a cause of debility and evomission proving directly debilitating. I cannot be mistaken in this, from the speedy restoration consequent upon the extrusion of this offensive tenant; and I am convinced that the health of our patient, which was, before he applied to me, deteriorating very rapidly, with imminent symptoms of hectic, constriction on the chest, dry cough, and flushing, nausea and morning pukings, obstipation, and cholicy pains in the abdomen, must have shortly become irreparable, but by removing the torpor of the alimentary canal with bitter purgatives, increasing the secretions with mercurial stimuli, exercise and nourishing light diet, and invigorating the habit with anti-asthenic remedies. The exfoliation and extrusion of unsound parts was at last effected, after many months had elapsed from the first discovery of the disease; and now, when the discharge is very slight, is inodorous and insipid to the patient, and what one might denominate muco-puriform rather than matter itself; this, conjoined with good health, may induce us to conclude the cure is complete, or not far off.

I beg pardon for detaining you so long on so rare a case, what I wished was chiefly to impress an idea of the feebleness of chirurgical aid alone, rather than to convey instruction, and never to forget to make the instruments of medicine operate from the centre to the circumference, in order to improve the art of chirurgical medicine, an art capable of much greater extension, and an object of the highest utility; for though Nature is of herself a blind guide, she is tractable and obedient to well ordered direction.

P. S. It may be needless to add, that mercurial frictions were ordered along the course of the nasal process of the maxillary bone, and that the loss of bone has left a large space in common to the sinus and nostril, opening just above the inferior turbinated bone; to restore the tone of the parts, I cause him to wash them, by making common water pass through the aperture and out again through the nostril in a stream, but no inconvenience results from this void at present, and no doubt it will shortly become of no importance when the parts consolidate.

